# Supplementary figures and images for: Evidence for gene-environment correlation in child feeding: Links between common genetic variation for BMI in children and parental feeding practices
Source: PLoS Genet. 2018 Nov 20;14(11):e1007757. doi: 10.1371/journal.pgen.1007757 (PMC6245504; doi:10.1371/journal.pgen.1007757)

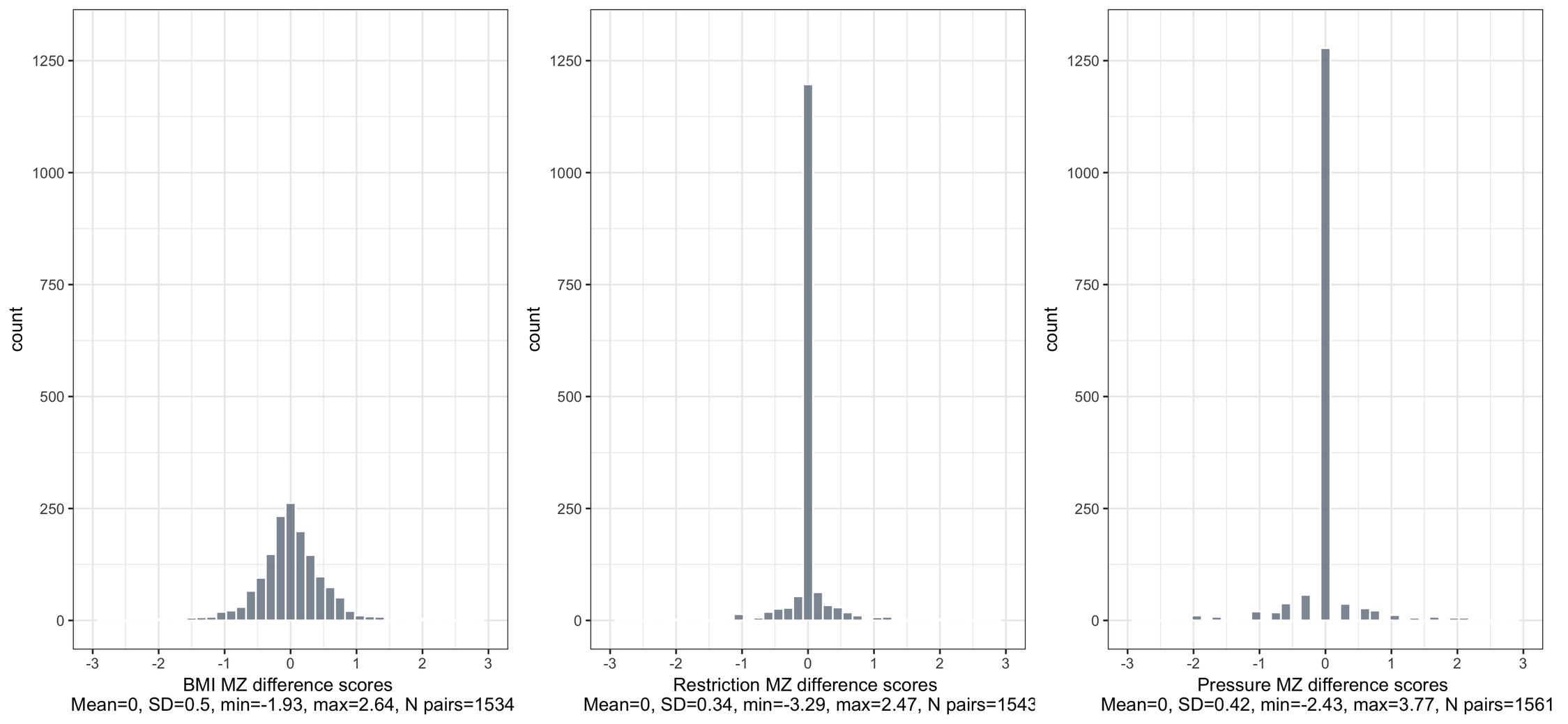

Supplement: S1 Fig — (TIF) [file pgen.1007757.s007.tif]

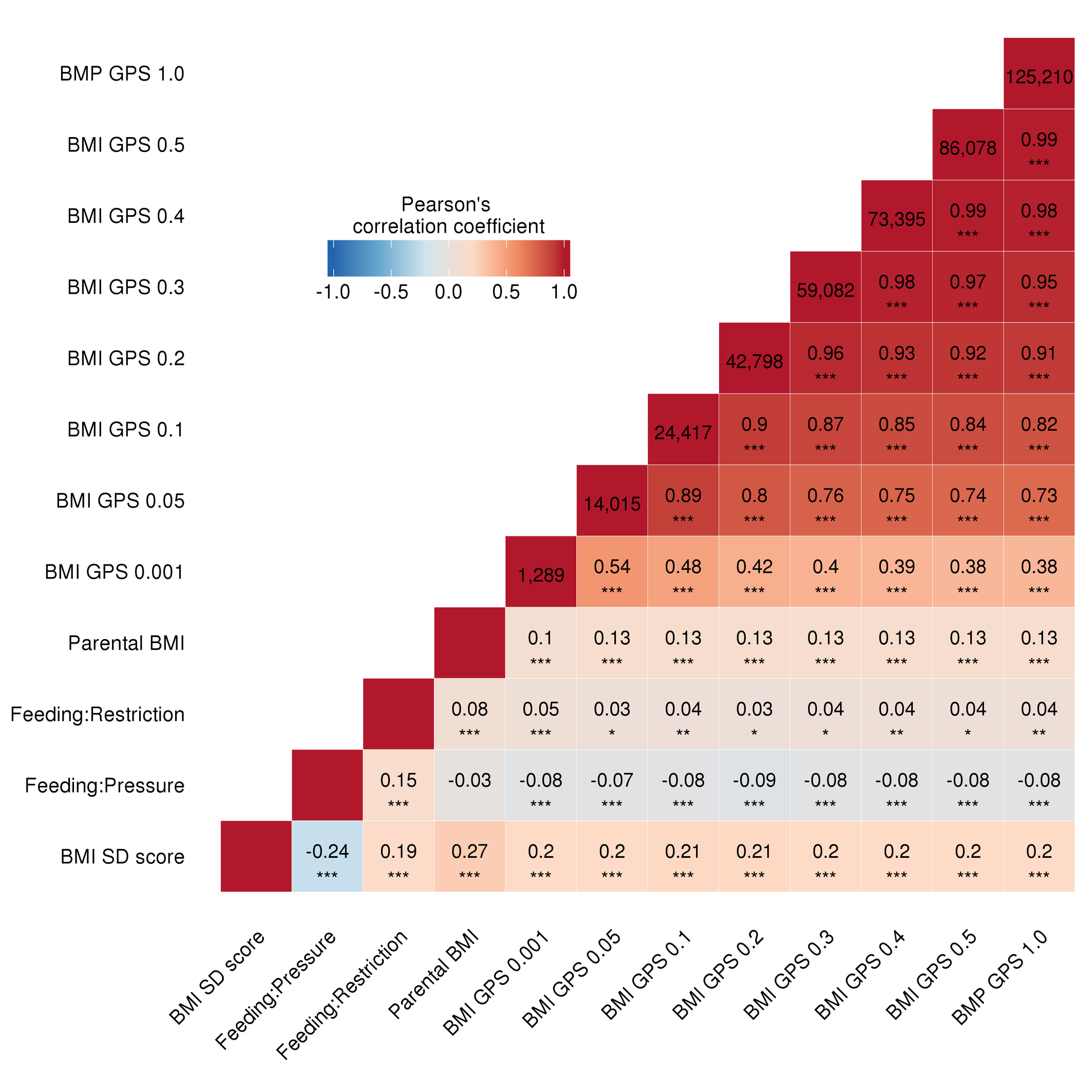

Supplement: S2 Fig — Diagonals of Genome-wide Polygenic Scores (GPS) show number of SNPs included in each respective score. * = p<0.05; ** = p<0.01; *** = p<0.001. (TIF) [file pgen.1007757.s008.tif]
